# Burns infection profile of Singapore: prevalence of multidrug-resistant *Acinetobacter baumannii* and the role of blood cultures

**DOI:** 10.1186/s41038-016-0038-8

**Published:** 2016-04-21

**Authors:** Christopher Tam Song, Jolie Hwee, Colin Song, Bien Keem Tan, Si Jack Chong

**Affiliations:** 1 University of Edinburgh, Edinburgh, UK; 2 Singapore General Hospital, Singapore, Singapore; 3 Cape Clinic Singapore, Singapore, Singapore

**Keywords:** Drug resistant, Burns, *Acinetobacter baumannii*, Blood cultures, Epidemiology, Singapore, Infection

## Abstract

**Background:**

With various changes implemented such as perioperative antibiotics for tangential excision, this retrospective study reviews the infection profile of burn patients at Singapore’s only centralized burns unit. Worldwide, the appearance of multidrug-resistant (MDR) strains of *Acinetobacter baumannii* (*A. baumannii*) continues to worsen patient outcomes. This study also surveys the role of blood cultures in burns at our unit.

**Methods:**

Four hundred fifty-two burn patients admitted to the unit between 2011 and 2013, and with cultures performed, were included in the study. The yields of various cultures were evaluated and 2684 samples were amassed, of which 984 (36.7 %) were positive. Patient variables for predictors of MDR *A. baumannii* infection acquisition and bacteremia were evaluated through multivariate analyses.

**Results:**

*Pseuodomonas aeruginosa* (*P. aeruginosa*) (67 patients) was the most common organism in those with total body surface area (TBSA) burn <20 % while MDR *A. baumannii (*39 patients) was most prevalent in those with TBSA burn ≥20 %. We found a yield of 1.1 % positive blood cultures for TBSA burn <20 % and a yield of 18.6 % positive cultures in TBSA burn ≥20 %. The median time between surgery and bacteremia was 6.5 days (range -18 to 68 days, interquartile range 4.5); 2.9 and 8.8 % of bacteremic episodes occurred within 24 and 48 h, respectively. This is a decrease from a predeceasing study (45.3 % for 24 h and 60 % for 48 h). Multivariate analysis revealed that length of hospital stay and TBSA burn ≥20 % were predictors of MDR *A. baumannii* infection and positive blood cultures.

**Conclusions:**

MDR *A. baumannii* infection burdens patient management, especially in those with TBSA burn ≥20 % and longer hospital stay. Prophylactic antibiotics may reduce perioperative bacteremia, but their role in MDR infections needs to be evaluated. The role of blood cultures in TBSA burn <20 % needs reconsideration.

## Background

The burns unit at Singapore General Hospital is the centralized service for the island nation and consistently reviews the infection profiles of Singapore’s burn patients. Acquiring multidrug resistant (MDR) *Acinetobacter baumannii* (*A. baumannii*) infection is associated with an increased risk of patient mortality, and outbreaks have led to the closure of wards [1]. The appearance of MDR strains of *A. baumannii* continues to rise and persists as a complication of burns worldwide [2]. Our unit strives to stay cognizant to the trends of culture results and antibiotics sensitivity, and this study is a comprehensive review of culture results of Singapore’s burn patients admitted between 2011 and 2013. This may be integral to the improvement of infection management in future patients.

## Methods

### Location

This study was conducted at the Singapore General Hospital Burns Unit, which is the sole facility for specialized burn care in the country of 5.5 million [3]. The unit consists of facilities dedicated to burn care such as an intensive care unit (ICU), a high dependency unit (HDU), an operating theater, wards, a skin lab, and a physiotherapy center.

### Design of study

**Fig. 1:**
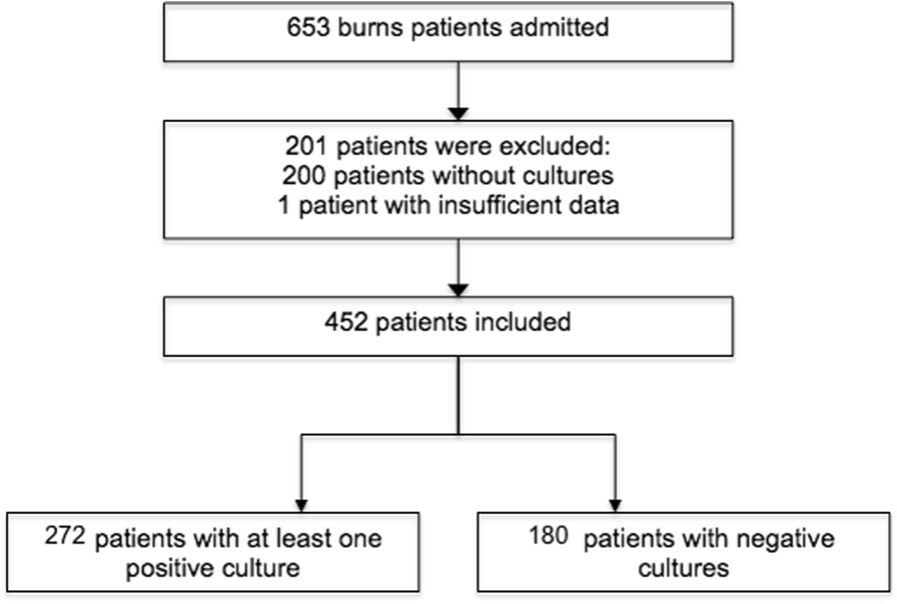
Patient selection process according to inclusion criteria for the study

The inclusion criterion for the study was admission to the unit, between January 2011 and December 2013, for burn-related injuries. Of the 653 burn patients admitted during this period, 201 patients did not have cultures performed and were excluded from this study (Fig. 1). A total of 2684 cultures were collected for all 452 patients.

This study aims to identify patient factors, specific to Singapore’s population, that are associated with positive MDR *A. baumannii* cultures or positive blood cultures. Both of these may signify poorer patient outcomes. Patients were categorized according to the percentage of total body surface area (TBSA) burn.

### Data collection

Data was collected from the patient records in the hospital burns database and was computed into a Microsoft Excel sheet. Data recorded included age, gender, nationality, TBSA burn, cause of burn, presence of inhalation injury intubation, length of stay, number of surgeries, and microbiology culture and sensitivity (type of culture, culture location, and multiple drug resistance status; defined as resistance to three or more antibiotics). The study attained institutional review board (IBR) approval from the Clinical Trials Resource Centre at Singapore General Hospital.

### Statistical analysis

Statistical analysis was performed using SPSS version 21.0 (SPSS, Chicago, IL). Student’s *t* test or the Mann–Whitney *U* test was used for continuous variables and chi-square test or Fisher’s exact test for categorical variables. A logistic regression model was used to identify risk factors for MDR *A. baumannii* infections. Odds ratios and 95 % confidence intervals were calculated. We also used parameters, with a *p* value <0.05 following univariate analysis, to further derive the best multiple binary logistic regression model.

## Results

### Burn infection epidemiology

Of the 452 patients who were screened for infection, 272 (60.2 %) were found to have positive cultures (Table 1). Each patient was categorized in five groups encompassing TBSA burn of less than 10 % (I), greater or equal to 10 % but less than 20 % (II), greater or equal to 20 % but less than 40 % (III), greater or equal to 40 % (IV), and exclusively inhalational injuries (V) (Table 1). Across the groups, as TBSA burn increases, there is a trend towards an increase across all patient factors illustrated, except patient age. In general, the most common organisms cultured overall were *Staphylococcus aureus* (*S. aureus*), *Pseuodomonas aeruginosa* (*P. aeruginosa*), and MDR *A. baumannii* (Table 2).

**Table 1:** Demographics of patients included in the study

		TBSA group				
Variables	Total	I	II	III	IV	V
Patients, *n* (%)	452 (100)	277 (61.3)	85 (18.8)	50 (11.1)	29 (6.4)	11(2.4)
Age, year						
Median	40	40	42	35.5	37	48
Range	5–94	14–94	18–83	54–82	5–70	5–70
*p* value	–	–	0.832	0.367	0.185	0.161
Sex						
Male, *n* (%)	255 (56.4)	151 (54.5)	43 (50.6)	35 (70.0)	23 (79.30)	3 (27.3)
*p* value	–	–	0.707	0.123	0.013	0.538
Nationality						
Foreign	176 (38.9)	84 (30.3)	41 (48.2)	31 (62.0)	18 (62.1)	2 (18.2)
*p* value	–	–	0.03	<0.001	<0.001	0.384
TBSA %						
Median	6.0	3.0	13.0	26.0	50.5	–
Range	0.1–90.0	0.1–9.5	10.0–19.5	20.0–38.0	40.0–90.0	–
Length of stay, day						
Median	11	10	13	23.5	27	6
Range	1–221	1–151	1–56	5–149	1–221	2–13
*p* value	–	–	<0.001	<0.001	0.013	0.582
Surgery, *n* (%)	365 (80.8)	222 (80.1)	73 (85.9)	45 (90.0)	25 (86.2)	0 (0)
*p* value	–	–	0.236	0.105	0.434	–
Patients with positive cultures, *n* (%)	272 (60.2)	172 (62.1)	35 (41.2)	36 (72.0)	25 (86.2)	4 (36.4)
*p* value	–	–	0.629	0.236	0.030	0.707

*TBSA* ​total body surface area, I TBSA burn < 10 %, II 10 % ≤ TBSA burn < 20 %, III 20 % ≤ TBSA burn < 40 %, IV TBSA burn ≥ 40 %, V Inhalational injury

**Table 2:** Incidence of each organism cultured in total population sample and according to TBSA group

Organism	Total infected, *n* (%)	I, *n* (%)	II, *n* (%)	III, *n* (%)	IV, *n* (%)	V, *n* (%)
Total no. of patients	452	277	85	50	29	11
*Pseudomonas aeruginosa*	80 (17.7)	51 (18.4)	8 (9.4)	11 (22.0)	10 (34.5)	0
*Staphylococcus aureus*	80 (17.7)	59 (21.3)	8 (9.4)	10 (20.0)	2 (6.9)	1 (9.1)
MDR *Acinetobacter baumannii*	54 (11.9)	8 (2.9)	6 (7.1)	17 (34)	22 (75.9)	1 (9.1)
MRSA	45 (10.0)	22 (7.9)	7 (8.2)	8 (16)	8 (27.6)	0
*Enterobacter* spp.	42 (9.3)	27 (9.7)	7 (8.2)	5 (10)	3 (10.3)	0
*Acinetobacter baumannii*	37 (8.2)	25 (9.0)	6 (7.1)	5 (10)	1 (3.4)	0
*Klebsiella* spp.	34 (7.5)	14 (5.1)	5 (5.9)	6 (12)	8 (27.6)	1 (9.1)
*Enterococcus* spp.	33 (7.3)	17 (6.1)	1 (1.2)	9 (18)	1 (3.4)	0
*Candida* spp.	30 (6.6)	10 (3.6)	1 (1.2)	8 (16)	10 (34.5)	1 (9.1)
MDR *Klebsiella* spp.	18 (4.0)	2 (0.7)	4 (4.7)	8 (16)	4 (13.8)	0
*Proteus mirabilis*	13 (2.9)	8 (2.9)	0 (0)	1 (2)	3 (10.3)	1 (9.1)
MDR *Staphylococcus aureus*	13 (2.9)	9 (3.2)	3 (3.5)	0 (0)	1 (3.4)	0
*Escherichia coli*	12 (2.7)	8 (2.9)	0 (0)	3 (6)	1 (3.4)	0
*Stenotrophomonas maltophilia*	11 (2.4)	5 (1.8)	1 (1.2)	1 (2)	4 (13.8)	0
MDR *Pseudomonas aeruginosa*	11 (2.4)	3 (1.1)	0 (0)	5 (10)	3 (10.3)	0
Group G *Streptococcus*	8 (1.8)	6 (2.2)	0 (0)	2 (4)	0 (0)	0
*Serratia* spp.	8 (1.8)	6 (2.2)	0 (0)	1 (2)	1 (3.4)	0
*Morganella morganii*	7 (1.5)	3 (1.1)	3 (3.5)	0 (0)	1 (3.4)	0
MDR coagulase-negative *Staphylococcus*	6 (1.3)	3 (1.1)	0 (0)	3 (6)	0 (0)	0
MDR *Enterobacter* spp.	6 (1.3)	4 (1.4)	0 (0)	1 (2)	1 (3.4)	0
Group A *Streptococcus*	5 (1.1)	4 (1.4)	0 (0)	1 (2)	0 (0)	0
Group B *Streptococcus*	5 (1.1)	5 (1.8)	0 (0)	0 (0)	0 (0)	0
MDR *Escherichia* c*oli*	4 (0.9)	0 (0)	1 (1.2)	0 (0)	3 (10.3)	0
MDR *Proteus mirabilis*	4 (0.9)	1 (0.4)	0 (0)	2 (4)	1 (3.4)	0
*Achromobacter xylosoxidans*	2 (0.4)	0 (0)	0 (0)	0 (0)	2 (6.9)	0
*Burkholderia cepacia*	2 (0.4)	0 (0)	0 (0)	2 (4)	0 (0)	0
*Staphylococcus lugdunensis*	2 (0.4)	1 (0.4)	1 (1.2)	0 (0)	0 (0)	0
*Trichosporon* spp.	2 (0.4)	1 (0.4)	0 (0)	1 (2)	0 (0)	0
*Aeromonas hydrophila*	1 (0.2)	1 (0.4)	0 (0)	0 (0)	0 (0)	0
*Arcanobacterium haemolyticum*	1 (0.2)	1 (0.4)	0 (0)	0 (0)	0 (0)	0
*Corynebacterium* spp.	1 (0.2)	1 (0.4)	0 (0)	0 (0)	0 (0)	0
*Clostridium* spp.	1 (0.2)	0 (0)	0 (0)	0 (0)	1 (3.4)	0
*Delftia acidovirans*	1 (0.2)	0 (0)	0 (0)	0 (0)	1 (3.4)	0
*Providencia* spp.	1(0.2)	0 (0)	1 (1.2)	0 (0)	0 (0)	0
MDR *Achromobacter xylosoxidans*	1(0.2)	0 (0)	0 (0)	0 (0)	0 (0)	1 (9.1)
MDR *Pseudomonas putida*	1 (0.2)	1 (0.4)	0 (0)	0 (0)	0 (0)	0

*TBSA* total burn serface area, *MDR* multidrug-resistant, *MRSA* Methicillin-resistant Staphylococcus aureus

The cumulative value of the total numbers of positive and negative for each culture type and the yields are shown (Table 3). In total, 984 cultures were positive out of the 2684, giving a general yield of 36.7 %. In descending order, the cumulative yield for each type of culture were 52.6 % for wound, 52.0 % for tissue, 39.3 % for endotracheal, 27.5 % for central line, 19.5 % for urine, and 14.2 % for blood. The percentage of patients with positive cultures, out of those tested, is 19.7 % (34 out of 173). Low-yield rates include line cultures for inhalational injury (0 %), blood culture for groups I (1.6 %) and II (0 %), and urine culture for group II (0 %). The three most common organisms found in the line cultures were MDR *A. baumannii* (9 patients), MDR *P. aeruginosa* (5 patients), and Methicillin-resistant Staphylococcus aureus (MRSA) (4 patients), respectively. Endotracheal cultures revealed MDR *A. baumannii* (21 patients), *Klebsiella* (7 patients), and *P. aeruginosa* (4 patients) as the most common organisms in intubated patients, accordingly. The mortality rate was 2.7 % for the study cohort (12 patients).

**Table 3:** Yield of the various types of culture in total and according to TBSA group

		TBSA group				
Culture	Total, n (%)	I, n (%)	II, n (%)	III, n (%)	IV, n (%)	V, n (%)
Wound	323	196	64	46	17	0
Yield	170 (52.6)	104 (53.1)	25 (39.1)	32 (69.6)	9 (52.9)	0
Tissue	1070	381	174	231	284	0
Yield	556 (52.0)	207 (54.3)	52 (29.9)	116 (50.2)	181 (63.7)	0
Endotracheal	163	29	6	33	86	9
Yield	64 (39.3)	8 (27.6)	2 (33.3)	15 (45.5)	36 (41.9)	3 (33.3)
Line	149	20	2	44	82	1
Yield	41 (27.5)	5 (25.0)	1 (50.0)	13 (29.5)	22 (26.8)	0
Urine	267	83	17	75	83	9
Yield	52 (19.5)	20 (24.1)	0 (0)	18 (24)	12 (14.5)	2 (22.2)
Blood	711	123	57	205	315	11
Yield	101 (14.2)	2 (1.6)	0 (0)	23 (11.2)	74 (23.5)	2 (18.2)

*TBSA* total body surface area, I TBSA burn < 10 %, II 10 % ≤ TBSA burn < 20 %, III 20 % ≤ TBSA burn < 40 %, IV TBSA burn ≥ 40 %, V inhalational injury

### MDR *A. baumannii* acquisition

Table 4 illustrates the antibiotic resistance profile of MDR *A. baumannii* isolates. MDR *A. baumannii* at our institution is defined as resistance to more than two classes of antibiotics. A total of 54 patients acquired MDR *A. baumannii*-positive cultures: 39 (72.2 %) of them presented with TBSA burn >20 % (groups III and IV). A univariate analysis of potential predictors for MDR *A. baumannii* was performed. Independent risk factors for MDR *A. baumannii* infection included length of stay, TBSA groups III and IV, intubation, and number of surgeries (Table 5). A multivariate analysis of these variables found that length of stay and TBSA groups III and IV were significant predictors of MDR *A. baumannii* acquisition (Table 6).

**Table 4:** Antibiotic resistance profile of multidrug-ressitant (MDR) *Acinetobacter baumannii* (A. baumannii) isolates

No.	Isolate	No. of cultures in each profile
1	Ceft + Pip/Taz + Amp	14
2	Ceft + Cep + Cefe + Ami	10
3	Amp + Cefe + Gen	6
4	Cep + Cip + Cot	5
5	Ceft + Mero + Erta	4
6	Cip + Imi + Min	3
7	Pip/Taz + Cep + Cefe	2
8	Amp + Cep + Cip + Min	2
9	Pip/Taz + Cep + Cot + Min	2
10	Ceft + Pip/Taz + Ami + Mer + Pol	2
11	Ceft + Cip + Cot + Gen + Ert	1
12	Ceft + Pip/Taz + Amp + Cepha + Cefe + Cip + Ami + Cot + Gen + Mero + Imi + Ert	1
	Total	54

*Ceft* ceftriaxone, *Pip/Taz* piperacillin/tazobactam, *Amp* ampicillin, *Cep* cephalothin, *Cefe* cefepime, *Cip* ciprofloxacin, *Ami* amikacin, *Cot* cotrimoxazole, *Gen* gentamicin, *Mer* meropenem, *Imi* imipenem, *Ert* ertapenem, *Min* minocycline, and *Pol* polymyxin B

**Table 5:** Univariate analysis of patient variables for acquisition of multidrug-ressitant (MDR) *Acinetobacter baumannii* (*A. baumannii*) infection

	Acquired MDR *A. baumannii* infection			Univariate analysis		
Variable	Yes (*n* = 54)	No (*n* = 398)	*p* value	OR	95 % CI	*p* value
Sex						
Male	36	219	0.11	–	–	–
Nationality						
Local	27	249	0.076	1.67	0.94–2.96	0.078
Age (years), mean±SD	44.3 ± 16.4	43.1 ± 17.4	0.629	–	–	–
Length of stay (days), mean±SD	45.8 ± 43.8	12.3 ± 8.7	<0.001	1.10	1.07–1.13	<0.001
TBSA group						
I	8	269	–	–	–	–
II	6	79	0.14	2.55	0.86–7.58	0.091
III	17	33	<0.001	17.32	6.94–43.24	<0.001
IV	22	7	<0.001	105.68	35.05–318.60	<0.001
V	1	10	<0.001	3.36	0.38–29.53	0.274
Intubation	26	42	<0.001	7.87	4.23–14.66	<0.001
Number of surgeries, mean±SD	3.4 ± 4.8	1.4 ± 1.9	0.03	1.23	1.11–1.35	<0.001
Prior infection						
*A. baumannii*	5	32	0.79	–	–	–
MRSA	9	36	0.79	–	–	–
*P. aeruginosa*	12	68	0.353	–	–	–
Cause of burn						
Scalding	8	132	0.001	0.35	0.16–0.76	0.008
Flame	8	81	–	0.68	0.31–1.50	0.339
Blast	14	40	–	3.13	1.57–6.25	0.001
Others	24	145	–	1.40	0.79–2.48	0.255
Days to admission (days), mean±SD	2.6 ± 5.7	4.9 ± 15.8	0.032	0.963	0.91–1.02	0.196

*MDR A. baumannii* multidrug-ressitant *Acinetobacter baumannii*, *OR* odds ratio, *CI* confidence interval, *TBSA* total body surface area, I TBSA burn < 10 %, II 10 % ≤ TBSA burn < 20 %, III 20 % ≤ TBSA burn < 40 %, IV TBSA burn ≥ 40 %, V inhalational injury, *MRSA* Methicillin-resistant Staphylococcus aureus

**Table 6:** Multivariate analysis of patient variables for acquisition of MDR *A. baumannii* infection

	Multivariate analysis		
Variable	OR	95 % CI	*p* value
Length of stay	1.09	1.0–1.13	<0.001
TBSA group			
III	5.16	1.77–15.03	0.003
IV	69.28	15.21–315.63	<0.001
Intubation	1.73	0.45–3.73	0.309
Number of surgeries	0.90	0.76–1.03	0.140
Cause of burn			
Blast	0.49	0.09–1.12	0.254

*MDR A. baumannii*  multidrug-ressitant *Acinetobacter baumannii*, *OR* odds ratio, *CI* confidence interval, *TBSA* total body surface area, III 20 % ≤ TBSA burn < 40 %, IV TBSA burn ≥ 40 %

### Blood culture

Thirty-four out of 173 patients were found to have positive blood cultures (19.7 %). Two of the 34 patients presented in group I, 11 in group III, 20 in group IV, and one in group V. There was a mortality of five patients with positive blood cultures (14.7 %) and five patients with negative blood cultures (3.6 %). The most common organism identified in the blood cultures was MDR *A. baumannii* in 15 patients (44.1 %), followed by *S. aureus* in six patients (17.6 %) and *P. aeruginosa* in six patients (17.6 %); 30 of the patients underwent surgery, giving a median of 6.5 days (range -18 to 68 days, interquartile range 4.5–9, Q1 = 4.5, Q3 = 9) to positive blood cultures. Positive cultures 24 h after surgery was found in one patient and 48 h in another two patients.

The univariate analysis of patient demographics found that age, foreign nationality, TBSA groups III and IV, flame as a cause of burn, a MDR *A. baumannii*-positive culture, length of stay, and number of surgeries were significant independent risk factors for a positive blood culture (Table 7). These factors were further analyzed with multivariate logistic regression that showed length of stay and TBSA burns groups III and IV were potential predictors for positive blood cultures (Table 8).

**Table 7:** Univariate analysis of patient variables for positive blood culture

	Blood culture			Univariate analysis		
Variables	Positive (*n* = 34)	Negative (*n* = 139)	*p* value	OR	95 % CI	*p* value
Age (years), mean±SD	38.1 ± 15.8	46.1 ± 16.0	0.010	0.97	0.94–0.99	0.011
Sex, n						
Male	26	88	0.147	1.89	0.79–4.47	0.151
Nationality, n						
Local	13	82	0.029	0.43	0.20–0.93	0.032
Foreigner	21	57	–	2.32	1.08–5.02	
TBSA, n						
I	2	61	<0.001	–	–	–
II	0	33	<0.001	0	0	0.998
III	11	31	0.220	10.82	2.26–51.89	0.003
IV	20	8	<0.001	76.25	14.94–389.10	<0.001
V	1	6	0.720	5.08	0.40–64.63	0.210
Cause, n						
Flame	15	29	0.029	3.00	1.36–6.61	0.007
Scalding	4	39	–	0.34	0.14–1.72	0.057
Blast	6	28	–	0.85	0.32–2.25	0.743
Others	9	43	–	0.80	0.35–1.87	0.611
MDR *A. baumannii* positive, n	18	30	0.001	4.09	1.86–8.97	<0.001
Length of stay, day	18.3 ± 16.3	53.9 ± 48.2	<0.001	1.05	1.03–1.07	<0.001
Days to admission, day	2.1 ± 4.3	2.5 ± 5.4	0.619	0.98	0.90–1.07	0.664
Days to first surgery, day	4.6 ± 6.9	5.5 ± 7.6	0.658	0.98	0.92–1.05	0.535
Number of surgeries	1.8 ± 1.8	6.2 ± 1.1	<0.001	1.44	1.22–1.70	<0.001

*OR* odds ratio, *CI* confidence interval, *TBSA* total body surface area, I TBSA burn < 10 %, II 10 % ≤ TBSA burn < 20 %, III 20 % ≤ TBSA burn < 40 %, IV TBSA burn ≥ 40 %, V inhalational injury, MDR* A. baumannii* multidrug-ressitant *Acinetobacter baumannii*

**Table 8:** Multivariate analysis of patient variables for positive blood culture

	Multivariate analysis		
Variables	OR	95 % CI	*p* value
Nationality			
Foreigner	1.82	0.47–7.15	0.388
TBSA group			
III	7.14	1.55–32.94	0.012
IV	51.58	7.52–353.78	<0.001
Cause			
Flame	1.66	0.41–6.76	0.482
Length of stay	1.03	1.00–1.06	0.022
Number of surgeries	1.11	0.86–1.44	0.421
MDR *A. baumannii*-positive culture	0.79	0.15–4.29	0.786

*OR* odds ratio, *CI* confidence interval, *TBSA* total body surface area, III 20 % ≤ TBSA burn < 40 %, IV TBSA burn ≥ 40 % *MDR A. baumannii* multidrug-ressitant Acinetobacter baumannii

## Discussion

### Epidemiology of burn infections in Singapore so far

This retrospective analysis found that in the burns population of Singapore, *S. aureus* and *P. aeruginosa* were the most common organisms cultured. Wong et al. [4] found that with a more selective criteria of >10 % burns and a minimum 7-day hospital stay, MDR *A. baumannii* presented with the highest prevalence in their cohort. Similar findings in the study of burn ICU patient infections are reported [5, 6]. Table 2 may explain the cause for difference in observations, being that the most common organism cultured in group I was *S. aureus* while in group IV this trended towards MDR *A. baumannii*. Various studies [7–9] have demonstrated this positive correlation between longer hospital stay with increased prevalence of *A. baumannii* infection. Upon acute admission, stratifying patients via TBSA burns, as with this study, may allow more straightforward means of quantifying the patient’s risk of infection.

### MDR *A. baumannii* prevalence

The care of the patients with burn wounds infected with MDR *A. baumannii* is challenging and carries a high mortality rate worldwide [10, 11]. Patients with prolonged hospitalization often become colonized, and the results of this study may aid anticipatory management in patients at high risk of MDR *A. baumannii* acquisition. Wong et al. [4] concluded that a prior MRSA infection was a risk factor to MDR *A. baumannii* infection. Contrary to this, the univariate analysis in Table 4 does not support the notion that *A. baumannii*-, MRSA-, or *P. aeruginosa*-positive cultures are independent risk factors for MDR *A. baumannii* acquisition. Wong et al. [4] also found that the number of intravascular lines placed and the Acute Physiology and Chronic Health Evaluation II score (APACHE II) on admission were significant predictive factors. The APACHE II score was not available for data collection, for the purpose of this study. However, the multivariate analysis in Table 6 supports this relationship with severe burns in ICU admissions, whereby groups III (OR, 5.16) and IV (OR, 69.28) and longer lengths of stay were significant predictors of MDR *A. baumannii*-positive cultures. Singapore employs a large foreign workforce that is involved in high-risk jobs that predisposes to more traumatic burn injuries. This may explain why a foreign nationality is an independent risk factor for MDR *A. baumannii* acquisition in Singapore.

Ward beds are implicated as reservoirs in MDR *A. baumannii* outbreak studies [12, 13]. In fact, an investigation used whole genome sequencing and traced an outbreak source to an operation theater and a bed [14]. The aerial dissemination of *A. baumannii* species presents the greatest challenge to decontaminative efforts [15]. In 2003, the unit implemented environmental and staff infection control measures including mandatory personal protective wear, hand hygiene, and terminal cleaning with hypochlorite. The beds are clear for use with three negative room samples while air is less than 60 % humidified and directed away from the patient with positive pressure airflow. A small prospective study found higher *Acinetobacter*- and *Enterobacter*-positive culture occurrences in negative pressure ventilation in the ward, while positive pressure was linked to higher prevalence of MRSA- and *Streptococcus*-positive cultures [16]. Barbut et al. [17] described an 88.8 % *A. baumannii* acquisition reduction with an infection control bundle. This included regular hydrogen perioxide vaporization disinfection, isolation of new admissions until proven negative cultures, and utilization of air purifiers. Another study [18] reported successful eradication of MDR *A. baumannii* after infection control interventions. The outbreak was linked to shower rooms that were found to be a colonization reservoir, and ironically, it was a common practice to shower all newly admitted patients. Their changes included improved patient showering practices, reduced use of low-concentration chlorhexidine, and cessation of occlusion dressings in third-degree burns.

### The role of blood cultures in burns so far

The American Burn Association [19] defines bloodstream infection by either of two criteria:

1. The patient’s blood cultures must identify a recognized pathogen in two or more separate instances or in one but with signs of sepsis.
2. Patient has a common skin contaminant cultured from two or more blood cultures drawn on separate occasions (including one drawn by venipuncture) and the patient has clinical signs of sepsis.

Chong et al. [5] and their study of burn ICU patients found that 45.3 % of bacteremia occurred within 24 h of surgery and 60 % of episodes occurred by 48 h. Moreover, 2.9 and 8.8 % of positive blood cultures in this cohort developed within 24 and 48 h, respectively. This reduction in postoperative bacteremia may be a direct effect of the unit’s transition towards empirical antimicrobial cover during burn excision and improved aseptic techniques. Tissue cultures are sent off and antibiotics are then directed according to culture sensitivity, available 24 to 48 h later. This principle is based on the widely accepted “intraoperative bacterial shower” theory [20].

The reduced perioperative bacteremia may further emphasize better ward infection control. It remains unclear if the intraoperative empirical antimicrobials may act as a double-edged sword and encourage patient susceptibility to MDR organisms on the ward ultimately. A systematic review found no evidence that perioperative systemic antibiotic prophylaxis, compared with placebo or another antibiotic, influenced any outcome variable [21].

Blood cultures in the burn patient are expensive, invasive, and burdened with false positive results. There is a high prevalence of fever in the burn presentations that is believed to be a result of the early inflammatory response, and this may explain the high negative culture yield in the lower TBSA groups of this study (less than 2 %). The incidence of bacteremia in the higher TBSA burn may also be higher than true. An analysis of false positive results in blood cultures found an increased likelihood in severe burns [22]. It is known that skin flora alterations occur in burns and the misdirected use of antibiotics from contaminated cultures may increase patient morbidity [23].

A study found that fever peaked at 38 to 96 h after the burn injury in children, regardless of bacteremia [24]. In the hospitalized population, the literature remains divided over the use of pyrexia and a raised white cell count in the acute setting as risk factors for bacteremia [25, 26]. Keen et al.’s case-control study found that an increased threshold for blood cultures did not compromise patient outcomes and have formulated a guideline [22].

### Limitations

The inclusion of all burn patients, including the burn ICU patients, creates a difficult comparison with previous burns infection studies. The study also did not include depth of burns or APACHE II scores, which were not available in the patient intranet database. The study was also not able to classify degree of bacterial invasion or acknowledge how many blood culture positives were transient since indications or antibiotic activity was not included. Lastly, the small population size of patients under group V may limit the clinical applicability of the data.

## Conclusions

This large-scale retrospective study identifies key factors that predict MDR *A. baumannii* acquisition and positive blood cultures, especially of those with TBSA burn above 20 % and in patients with long lengths of stay. Perioperative broad-spectrum antibiotics may have reduced surgical bacteremia, but its role in acquisition of MDR infections warrants evaluation in future studies. The study also stimulates the need to scrutinize the necessity of blood cultures in patients with less than 20 % TBSA burn.
